# Knowledge Sharing Mediates the Relationship Between Nurses' Job Well‐Being and InnovativeBehaviour: A Cross‐Sectional Study

**DOI:** 10.1002/nop2.70602

**Published:** 2026-05-15

**Authors:** Qi Zhou, Yuqi Xu, Jiayu Zhang, Na Zhang, Junwei Ye, Qinrou Yu, Wenqing Guan, Yueli Zhu

**Affiliations:** ^1^ Hangzhou Linping District Hospital of Integrated Traditional Chinese and Western Medicine Hangzhou China; ^2^ Nursing Department Hangzhou Normal University Hangzhou Zhejiang Province China; ^3^ Lishui University Lishui Zhejiang Province China

**Keywords:** innovative behaviour, job well‐being, knowledge sharing, nurses

## Abstract

**Objectives:**

To examine whether nurses’ job well‐being is associated with their innovative behaviour and whether knowledge sharing mediates that relationship.

**Design:**

A cross‐sectional study.

**Method:**

This study used convenience sampling to select 1,025 nurses from 8 hospitals. The data was collected between June and November 2023 using the Nurses Job Well‐being Scale, Nurses Innovative Behaviour Scale, and Knowledge Sharing Scale. The data analysis process included descriptive statistics, correlation analysis, multivariate regression, and hypothesis testing.

**Results:**

Job well‐being was positively correlated with innovative behaviour (*r* = 0.63, *p* < 0.001) and with knowledge sharing (*r* = 0.73, *p* < 0.001). Knowledge sharing was also significantly associated with innovative behaviour (*r* = 0.55, *p* < 0.001). Regression analysis indicated that both job well‐being (β = 0.38, *p* < 0.001) and knowledge sharing (β = 0.23, *p* < 0.001) significantly predicted innovativebehaviour. Mediation analysis showed a significant indirect effect of job well‐being on innovative behaviour through knowledge sharing (indirect effect = 0.52, 95% CI [0.44, 0.60]).

**Conclusion:**

Nurses' job well‐being promotes innovative behaviour both directly and indirectly through knowledge sharing. Interventions targeting psychological well‐being and structured knowledge‐sharing mechanisms may enhance innovation in nursing practice.

**Patient or Public Contribution:**

This study did not involve patients or the public in the design, conduct, or reporting of the research.

## 1 Introduction

As the medical paradigm evolves and care delivery expands, society's need for health care is growing. Because nurses' roles extend beyond clinical duties, it is imperative to adopt innovative approaches to address existing or potential health problems (Zhang and Wang 2015). Innovative behaviour involves generating novel ideas, securing support, and implementing new practices in clinical work, including producing, championing, and realising ideas (Ling et al. 2012). This behaviour encompasses the introduction of innovations in clinical nursing practice, education, and research. Researchers have shown that both internal and external factors influence and constrain nurses' innovative behaviour (Hong et al. 2018; Jalali and Heidari 2016; Jing 2019; Nguyen et al. 2020; Liu 2021). However, research has primarily emphasised external organisational factors while overlooking internal factors, such as individual cognitive assessment and affective components.

Job well‐being refers to nurses' positive work‐related affect and cognitive evaluations of their job, reflecting sustained positive psychological experiences at work (Chen and Liu 2016). Positive psychology perspectives emphasise how positive affect broadens cognitive repertoires and builds psychological resources, helping explain links between well‐being and creativebehaviour. At the same time, organisational factors shape job well‐being and its outcomes (Hao et al. 2018; Tang and Lv 2019; Chen and Liu 2022). Empirical studies across organisational contexts have linked job well‐being with motivation, engagement, and creative or innovative performance (Koroglu and Ozmen 2022; Putra and Pramusiwi 2023). However, much of this evidence comes from corporate or mixed‐occupational samples; fewer studies have examined these pathways in nursing. Therefore, nurse‐centred research is needed to determine whether these relationships hold in clinical settings and to clarify how knowledge sharing may operate as a mechanism. Nurses may engage in innovative behaviours as a strategy to alter unfavourable work conditions (Binnewies and Wörnlein 2011; Linton et al. 2021). Third, the mechanisms linking job well‐being to innovative behaviour remain underexplored, particularly in nursing settings. Although knowledge sharing has been proposed as a key behavioural pathway, its mediating role has not been rigorously tested among nurses. Addressing these gaps is essential to clarify how psychological and social processes jointly influence innovation in healthcare settings. The role of knowledge sharing needs to be emphasised in the pathway. Both individuals and organisations require knowledge as a foundation for innovative behaviour (Yi 2005). Knowledge sharing is conceptualised as a behavioural mechanism through which individual psychological states influence organisational outcomes (Ali et al. 2019; Michna and Kmieciak 2020; Shaari et al. 2015; Takhsha et al. 2020; Ju and Zhao 2020). According to social exchange theory, employees who experience positive psychological states are more willing to reciprocate through cooperative behaviours such as knowledge exchange.

Furthermore, positive affect enhances openness, cognitive flexibility, and collaboration, thereby facilitating knowledge‐sharingbehaviours. In turn, knowledge sharing provides access to diverse perspectives and informational resources, which are critical antecedents of innovativebehaviour. Therefore, knowledge sharing is proposed as a mediating pathway linking nurses' job well‐being and innovative behaviour (Nawaz et al. 2024). Several studies found that job well‐being promotes knowledge sharing. Knowledge sharing also promotes individual innovativebehaviour. Nevertheless, studies in the nursing profession have yet to definitively demonstrate a correlation among nurses' job well‐being, innovativebehaviour, and knowledge sharing. Furthermore, the mediating role of knowledge sharing has yet to be argued. Therefore, this study will verify the mediating role of knowledge sharing in the path.

This study examines the relationships among nurses' job well‐being, innovativebehaviour, and knowledge sharing. Theoretically, positive work‐related affect broadens attentional scope and builds cognitive and social resources that support creativity (Fredrickson 2004). At the organisational level, knowledge sharing provides the informational inputs and social endorsement that transform individual ideas into implemented innovations (Wang and Noe 2010). Therefore, job well‐being should increase nurses' propensity to share knowledge, which in turn facilitates innovativebehaviour; this mediational logic underpins H3. Therefore, we propose the following research question and hypotheses:

Research question:

Do nurses' job well‐being influence their innovativebehaviour, and is this relationship mediated by knowledge sharing?

We hypothesise that:

*Nurses’ job well‐being is positively associated with innovativebehaviour*.

*Nurses’ knowledge sharing is positively associated with innovativebehaviour*.

*Knowledge sharing mediates the relationship between nurses’ job well‐being and innovativebehaviour*.

## 2 Methods

We used convenience sampling to recruit nurses from eight hospitals across the western, central, and eastern regions, as reported in the 2019 China Health Statistics Yearbook. Convenience sampling is a pragmatic approach commonly used in clinical cross‐sectional studies when comprehensive sampling frames are unavailable (Shorten and Moorley 2014). We chose this method because of logistical constraints (access across multiple hospitals) and the study's exploratory aims; we acknowledge the trade‐offs in representativeness and address them in the limitations (Cathala and Moorley 2018).

### 2.1 Participants

Inclusion criteria: (1) Registered/licensed nurses currently employed in direct patient care; (2) at least one year of clinical nursing experience; (3) able to read the questionnaire language and willing to provide informed consent.

Exclusion criteria: (1) Nurses on extended leave (e.g., maternity, sick leave) during the survey window; (2) rotating trainees or those temporarily assigned to nonclinical roles; (3) participants with incomplete questionnaires (> 20% missing).

Sample size was calculated using G*Power 3.1 for multiple regression analysis with three predictors. Assuming a medium effect size (f^2^ = 0.15), a significance level of α = 0.05, and statistical power of 0.80 (Gülkesen et al. 2022), the minimum required sample size was 293. To account for potential non‐response or incomplete data, the sample size was increased by 15%.

### 2.2 Research Tools

Key variables: Independent variable is nurses' job well‐being (Nurses' Job Well‐Being Scale); Mediator is knowledge sharing (Knowledge Sharing Scale); Dependent variable is nurses' innovative behaviour (Nurse Innovative Behaviour Scale). Control variables included demographic and job characteristics:Age, gender, nursing tenure, education level, clinical department, night‐shift frequency, and hospital region/class.

Nurse InnovativeBehaviour: Ling Bao's 10‐item Nurses' Innovative Behaviour Scale (Ling et al. 2012). The scale includes three dimensions: Nurses producing ideas, receiving support, and realising ideas. The scale is a 5‐point Likert scale, ranging from severely non‐compliant to compliant. The scale ranges from 10 to 30, with higher scores indicating more innovative behaviour (Cronbach's α = 0.88).

#### 2.2.1 Nurses' Job Well‐Being

Chen Ling's 19‐item Nurses' Job Well‐Being Scale (Chen and Liu 2016). The scale includes five dimensions: Welfare benefits, interpersonal relationships, work value, managers, and job features. It is based on a 6‐point Likert scale ranging from strongly disagree to agree strongly. Scores range from 19 to 114, with higher scores indicating more job Well‐Being (Cronbach's α = 0.91).

#### 2.2.2 Knowledge Sharing Scale

Eight items; two subscales: Knowledge gathering and knowledge contribution (Wang 2016). Items rated 1 (strongly disagree) to 5 (strongly agree); possible scores 8–40. Higher scores indicate greater knowledge sharing (Cronbach's α = 0.95).

### 2.3 Data Analysis

All instruments used in this study had previously been translated and validated in Chinese nursing populations, demonstrating acceptable reliability and construct validity. Cronbach's α coefficients in the present study ranged from 0.88 to 0.94. Construct validity was assessed using confirmatory factor analysis. The measurement model demonstrated acceptable fit (CFI = 0.93, TLI = 0.92, RMSEA = 0.06). Composite reliability values ranged from 0.89 to 0.95, and average variance extracted (AVE) values exceeded 0.50 for all constructs, indicating satisfactory convergent validity. Control variables (age, gender, tenure, education level, department, night‐shift frequency, and hospital characteristics) were entered in the first step of hierarchical regression analyses to control for potential confounding effects. In the structural equation model, these variables were specified as covariates influencing innovativebehaviour. The study was statistically analysed using SPSS 26.0 and AMOS 24.0. Before analysis, assumptions of normality and linearity were assessed using skewness and kurtosis statistics. Multicollinearity was evaluated using variance inflation factors (VIF), with all values below 5 indicating no significant collinearity. Mediation analysis was conducted using bootstrap resampling (5000 iterations) to estimate bias‐corrected 95% confidence intervals. The data analysis process included descriptive statistics, correlation analysis, multivariate regression, and hypothesis testing. Results were statistically significant when *p <* 0.05.

### 2.4 Ethical Considerations

This study was approved by the Institutional Review Board of Hangzhou Normal University (Approval no. 2023040). The study's adherence to ethical principles will ultimately protect the participants' rights and privacy. This study followed the STROBE Guidelines.

## 3 Results

### 3.1 Results of Data Collection

Data were collected between June and November 2023. Trained research assistants visited participating hospital wards to invite eligible nurses, distribute paper questionnaires, and provide an anonymised online survey link for those unable to complete paper forms. In total, 1100 questionnaires were distributed across eight hospitals; after screening, 1025 valid questionnaires were retained for analysis (response rate 93.2%). Harman's single‐factor test revealed that the first unrotated factor accounted for 38% of the total variance, below the critical threshold of 40%, suggesting that common method bias is unlikely to significantly influence the results.

### 3.2 Results of Descriptive Statistics

Tertiary hospitals in the east were predominant. A total of 1025 nurses participated in the study, most of whom were women (88.5%). Table 1 presents detailed demographic and professional characteristics. The mean scores for job well‐being, knowledge sharing, and Innovative behaviour were 4.24 ± 0.89, 3.76 ± 0.73, and 3.68 ± 0.75.

**TABLE 1:** Demographic and professional characteristics.

Variable	Content	Number	Percentage (%)
Region	East	523	51.0
	Centre	317	31.0
	West	185	18.0
Hospital Class	Tertiary Hospitals	679	66.2
	Level II hospitals	177	17.2
	Level 1 hospitals	169	16.5
Gender	Male	118	11.5
	Female	907	88.5
Age	< 26	559	54.5
	26–36	403	39.3
	> 36	63	6.2
Work Experience	< 6	718	70.0
	6–11	191	18.6
	> 11	116	11.4
Department	Internal Medicine	157	15.3
	Surgery	134	13.1
	Obstetrics and Gynaecology	157	15.3
	Emergency Medicine	115	11.2
	ICU	125	12.2
	Operating theatre	105	10.2
	Outpatient	108	10.5
	Other Departments	124	12.1
Position	Junior Nurse	519	50.6
	Nurse Practitioner	349	34.0
	Supervisory Nurse	118	11.5
	Associate Nurse Practitioner and above	39	3.8
Education	College and below	217	21.2
	Bachelor's Degree	605	59.0
	Master and above	203	19.8
Marriage	Married	306	29.9
	Unmarried	696	67.9
	Divorced	23	2.2
Salary (Monthly)	< 6,000	532	51.9
	6,000–10,000	344	33.6
	> 10,000	149	14.5
Night Shifts (Monthly)	< 4	484	47.2
	4–8	375	36.6
	> 8	166	16.2
Scientific Research	Yes	222	21.7
	No	803	78.3
Job Satisfaction	Likes	287	28.0
	Middle	560	54.6
	Dislike	178	17.4

### 3.3 Results of Correlation Analysis

Pearson's correlation analysis revealed that job well‐being was positively correlated with both Innovative behaviour (*r* = 0.63, *p* < 0.001) and knowledge sharing (*r* = 0.73, *p* < 0.001). Moreover, knowledge sharing positively and significantly correlates with Innovative behaviour (*r* = 0.55, *p* < 0.001).

### 3.4 Results of Multivariate Regression Analysis

Standardised regression coefficients (*β*) along with 95% confidence intervals were reported to facilitate interpretation and comparison across variables. The multivariate regression analysis highlighted the significant impact of knowledge sharing and job well‐being on innovative behaviour (KS = 0.23, *p* < 0.001; JW = 0.38, *p* < 0.001), as shown in Table 2.

**TABLE 2:** Results of multivariate regression analysis.

Variable	Standardised coefficient		Standardised coefficient	Significance	VIF
	B	SE			
Constant	2.02	0.23		< 0.001	
Title	−0.01	0.03	0.01	0.74	1.53
Research Achievement	−0.37	0.07	−0.20	< 0.001	1.19
Work Attitude	−0.03	0.04	−0.02	0.51	1.15
Monthly salary	−0.07	0.04	−0.07	0.09	1.44
Knowledge sharing	0.23	0.05	0.22	< 0.001	2.21
Job well‐being	0.39	0.04	0.45	< 0.001	2.29

### 3.5 Results of the Main Effects Test and Bootstrap Test

**FIGURE 1:**
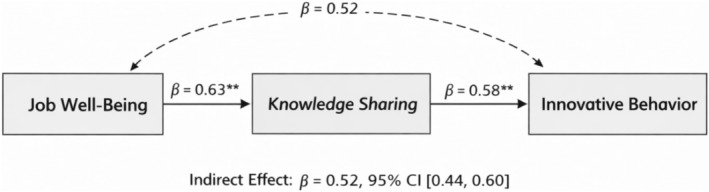
The mediation model with standardised path coefficients.

Chi‐Square = 2.99; Root Mean Square Error of Approximation = 0.06; Normed fit index = 0.98; Relative Fit Index = 0.97; Incremental Fit Index = 0.98; Tucker‐Lewis Index = 0.98; Goodness of fit index = 0.97; Comparative fit index = 0.98; shows the relationships among the factors in the fitting model. Job well‐being had an indirect impact of 0.52 on innovativebehaviour, as shown in Figure 1. The total effect of job well‐being on innovative behaviour was significant. After including knowledge sharing, the direct effect remained significant, indicating partial mediation. The indirect effect (0.52, 95% CI [0.44, 0.60]) accounted for a substantial proportion of the total effect, suggesting that knowledge sharing is a key explanatory mechanism, as shown in Table 3.

**TABLE 3:** The result ofthe bootstrap test.

Path	Effect	SE	*P*	PErcentile 95% CI	BIas‐corrected 95% CI
Job well‐being to Innovative behaviour	0.41	0.49	< 0.001	[0.314, 0.502]	[0.314, 0.501]
The Mediating Role of Knowledge Sharing in Job Well‐being to Innovative Behaviour	0.53	0.40	< 0.001	[0.447, 0.602]	[0.446, 0.601]

## 4 Discussion

This study examined nurses' innovative behaviour and its associations with job well‐being and knowledge sharing. The level of innovative behaviour observed in this study was broadly comparable to previous findings, although some differences were noted across studies (Yang et al. 2022; Ya et al. 2017). These variations may be related to differences in institutional contexts, professional development opportunities, and the increasing emphasis on innovation in nursing practice. With advances in healthcare systems and policies that encourage innovation, nurses may have more opportunities to engage in idea generation and implementation. Therefore, nursing managers may consider supporting innovative behaviour through training, appropriate incentives, and organisational support (Hong et al. 2018; Mengliang et al. 2023; Qing et al. 2023; Yannan et al. 2023).

Consistent with prior research, job well‐being was positively associated with innovative behaviour (Liu 2021; Hualiang et al. 2019; Wright and Hobfoll 2004). Nurses with higher job well‐being may be more likely to engage in positive work‐relatedbehaviours, including creative thinking and problem‐solving. From a theoretical perspective, positive psychological states may broaden cognitive resources and facilitate engagement in innovative activities (Wang and Noe 2010). However, some studies suggest that lower levels of job well‐being may also stimulate innovation through compensatory mechanisms (Yuan and Woodman 2010; Binnewies and Wörnlein 2011; Linton et al. 2021). The findings of this study are more consistent with the positive pathway, although such alternative explanations cannot be excluded and warrant further investigation.

The results also showed that job well‐being was positively associated with knowledge sharing, and that knowledge sharing was positively associated with innovativebehaviour, consistent with previous studies (Liu 2021; Guodong et al. 2019; Yang and Yong 2012). Nurses with higher job well‐being may be more willing to share their knowledge, skills, and experiences, partly due to increased motivation and a greater sense of trust and engagement in their work environment. In turn, knowledge sharing facilitates access to diverse perspectives and practical experience, which may support the development and implementation of innovative ideas. In healthcare settings, where teamwork and collaboration are essential, knowledge exchange may play a particularly important role in shaping innovative behaviour (Yi 2005; Wang and Noe 2010).

Importantly, this study extends existing research by examining the mediating role of knowledge sharing. The results indicated a significant indirect effect of job well‐being on innovative behaviour through knowledge sharing, suggesting that knowledge sharing may function as a key behavioural mechanism linking psychological well‐being and innovation (Nawaz et al. 2024). At the same time, the direct association between job well‐being and innovative behaviour remained significant after accounting for the mediator, indicating partial mediation (Jiaoyan et al. 2023). This suggests that job well‐being may be associated with innovative behaviour through both direct and indirect pathways.

Compared with previous studies conducted in non‐clinical or mixed samples, the mediating effect observed in this study appears relatively substantial. This may be related to the collaborative nature of nursing work, where knowledge sharing is embedded in daily clinical practice. In such contexts, the translation of psychological states into innovative behaviour may depend more strongly on interpersonal and team‐based processes. However, this interpretation should be approached with caution, as differences in measurement and study design may also contribute to variations in effect size.

From a practical perspective, the findings suggest that nursing management may benefit from a simultaneous focus on psychological well‐being and knowledge‐sharing practices. For example, maintaining manageable workloads and providing psychological support may help sustain nurses' job well‐being (Cai et al. 2023; Lv et al. 2019), while structured knowledge‐sharing approaches, such as mentoring systems, case discussions, and digital communication platforms, may facilitate the exchange of clinical knowledge (Jiaoyan et al. 2023). Recognition and encouragement from leadership may further promote engagement in both knowledge sharing and innovation (Mengliang et al. 2023). These implications should be considered exploratory and require further validation.

Several limitations should be acknowledged. First, the cross‐sectional design limits causal inference, and the findings should be interpreted as associations consistent with a mediation model rather than definitive causal relationships. Reverse or reciprocal relationships cannot be ruled out. Second, contextual factors such as leadership style or organisational climate were not included and may influence the observed associations. Third, the use of convenience sampling may limit generalisability. Future research should adopt longitudinal or experimental designs to clarify causal pathways and examine additional mediating or moderating mechanisms. Multi‐centre and cross‐cultural studies may further enhance the robustness and applicability of the findings.

## 5 Conclusion

Nurses' job well‐being was positively associated with innovativebehaviour, with knowledge sharing acting as a partial mediator. These findings suggest that knowledge sharing may be an important pathway linking well‐being and innovation. Practically, nursing leaders should protect nurses' well‐being through manageable workloads and accessible mental‐health supports, institutionalise knowledge‐sharing mechanisms (formal mentoring, interdisciplinary case conferences, digital collaboration platforms), and recognise and reward innovation. Future research should test these relationships longitudinally, include multi‐source outcome data, and examine cross‐cultural generalisability.

## Funding

The authors have nothing to report.

## Ethics Statement

The Institutional Review Board of China (Hangzhou Normal University) granted ethical approval. We explained the purpose of the study to the participants before distributing the questionnaire. In addition, we ensured that participants were voluntary and anonymous and allowed participants to opt out at any time. We stored the data securely, and only research team members could access, store, and process the data.

## Conflicts of Interest

The authors declare no conflicts of interest.

## Data Availability

The data that support the findings of this study are available from the corresponding author upon reasonable request.
